# Antibacterial resistances in uncomplicated urinary tract infections in women: ECO·SENS II data from primary health care in Austria

**DOI:** 10.1186/1471-2334-12-222

**Published:** 2012-09-18

**Authors:** Gustav Kamenski, Gernot Wagner, Sonja Zehetmayer, Waltraud Fink, Wolfgang Spiegel, Kathryn Hoffmann

**Affiliations:** 1 Karl Landsteiner Institute for Systematic in General Practice, Ollersbachgasse 144, 2261, Angern, Austria; 2 Department of General Practice, Centre for Public Health, Medical University of Vienna, Währingerstrasse 13a/III, 1090, Vienna, Austria; 3 Department of Medical Statistics, Medical University Vienna, Spitalgasse 23, 1090, Vienna, Austria

**Keywords:** Uncomplicated urinary tract infection, Primary health care, Austria, Antibiotic resistance

## Abstract

**Background:**

Uncomplicated urinary tract infections (UTI) are a frequent reason for consultation of women in primary health care. To avoid therapy failure and development of resistances, the choice of an antibiotic should be based on the knowledge of recent local resistance data but these data are scarce for the Austrian primary health care sector. Within the context of the ECO·SENS II study it was the aim to obtain appropriate and relevant local resistance data and describe the changes in the resistance pattern in comparison to the ECO·SENS study.

**Methods:**

23 GPs from different parts of Austria participated in the study between July 2007 and November 2008. According to the defined inclusion- and exclusion criteria female patients with symptoms of an uncomplicated UTI were included and a midstream urine sample was collected. In case of significant bacteriuria susceptibility testing of *E. coli* against 14 antibiotics was performed. Descriptive statistical methods were used.

**Results:**

In 313 patients included in the study, a total of 147 *E. coli* isolates (47%) were detected and tested. The resistance rates were in %: Mecillinam (0.0), nitrofurantoin (0.7), fosfomycin trometamol (0.7), gentamycin (1.4), cefotaxime (2.7), ceftazidime (2.7), Cephadroxil (4.1) and ciprofloxacin (4.1). Higher resistance rates were found in amoxicillin/clavulanic acid (8.9), nalidixic acid (9.6), trimethoprim/sulphamethoxazole (14.4), trimethoprim (15.8), sulphamethoxazole (21.2) and ampicillin (28.8). Additionally, the comparison of these results with the results of the ECO·SENS study demonstrated an increase in resistance rates of ampicillin, amoxicillin/clavulanic acid, nalidixic acid and ciprofloxacin.

**Conclusions:**

The resistance data for *E. coli* in uncomplicated UTIs in women gained by this study are the most recent data for this disease in Austria at the moment. The increased resistance rates of amoxicillin/clavulanic acid, ciprofloxacin and nalidixic acid should be respected when choosing an appropriate antibiotic for uncomplicated UTIs. The use of ampicillin, sulphamethoxazole, trimethoprim and trimethoprim/sulphametoxazole in uncomplicated UTIs in women should be questioned at all. The findings of this study should result in a regular surveillance system of resistances emerging in the ambulatory sector designed after the model of the EARS-Net.

## Background

Urinary tract infections (UTI) are a frequent reason for consultation of women in primary health care [1-4]. According to epidemiological surveys an annual incidence of 11.3 million cases in the USA and 175 million cases worldwide can be estimated [5,6]. Approximately 80% of the cases with a significant positive urine culture for pathogens an infection with *Escherichia coli (E. coli)* can be expected; other causative bacteria are *Staphylococcus saprophytic us*, *Klebsiella pneumonia*, *Proteus mirabilis* and group B streptococci [7,8].

The natural course of these UTIs shows a low rate of spontaneous cure even after a follow up of 6 weeks [9]. Since UTIs are a frequent reason for prescribing antibiotics (AB) the decision which AB should be prescribed has to be considered prudently. Normally, when antibiotics for treating an acute UTI are prescribed in primary health care the nature of the causative bacterium can only be presumed because current resistance data from the primary health care sector are scarce [10-12]. However, to avoid the increasing threat of antibiotic resistances the local resistance patterns, especially for UTIs where resistances to commonly used antibiotics like ampicillin and trimethoprim are increasing, have to be respected [13-17]. Therefore, it is necessary to gain susceptibility data especially from the primary health care sector and not only from hospitals or specialized centre’s because these resistances differ from that seen in the ambulatory sector [18].

Some studies on this topic have been performed. One has been the ECO·SENS project [17]. It was an international survey to investigate the prevalence and susceptibility of uropathogens causing acute uncomplicated community-acquired UTIs in primary care in the years 1999 and 2000. Collectively, 4,734 women with symptoms of acute UTI from 252 community care centre’s in 16 European countries including Austria and, in addition, Canada were enrolled. A total of 3,278 women (69.2%) had culture proven lower UTIs with *E. coli* responsible for 77% of all culture proven pathogens. Six years later a similar project, ECO·SENS II was conducted to provide and follow up data on epidemiology and susceptibility of uropathogens causing UTIs in primary care in 5 European countries: United Kingdom, Sweden, Austria, Portugal and Greece. In the context of the ECO·SENS II study it is the aim of the present analysis to provide data about the antimicrobial susceptibility of *E. coli* and *K. pneumonia* causing uncomplicated UTIs in women in primary health care in Austria and to compare the corresponding data of the ECO·SENS project (1999-2000) with the ECO·SENS II project (2007-2008) data. In addition, these results were contrasted with the resistance data for *E. coli* in the Austrian resistance report 2008 (AURES) for the ambulatory sector [15].

## Methods

### Patients and investigational centre’s

This study was designed as a cross-sectional study. During the study period from July 2007 to November 2008, all female patients aged 18- 65 years that consulted one of their GPs in 23 GP offices (investigational centre’s) with symptoms of uncomplicated UTI were included consecutively, if they met the inclusion criteria and agreed to sign the informed consent form. The investigational centre’s that had to subscribe an informed consent form too, were located in three geographical regions in Austria each separated from the other by at least 150 km. The geographical regions included larger cities as well as smaller cities and rural areas.

From the ECO·SENS study it was known that approximately 70% of the subjects included had an infection with uropathogens of which approximately 77% were *E. coli*. Therefore, it was the aim of the investigational centre’s to include 400 subjects to ensure that 200 isolates of *E. coli* were collected.

### Inclusion and exclusion criteria

Inclusion criteria: Female patients aged 18- 65 years with uncomplicated UTI.

After adequate information and signing the informed consent forms, the patients were asked to assess the presence and severity of 4 typical symptoms of an uncomplicated UTI: Frequency, urgency, dysuria and suprapubic pain. Presence and severity was assessed as follows: 0 = absent, 1 = mild, 2 = moderate and 3 = severe. The sum of the 4 symptoms gave the total symptom score (range 0-12). Only patients with a symptom score of ≥ 2 were included in the study.

Exclusion criteria: Symptoms lasting more than 7 days, signs of infection of the upper urinary tract, known structural or functional abnormalities of the urinary tract, indwelling urinary catheter or chronic incontinence, subjects who had received any kind of antibiotics within the previous 2 weeks for UTI or any other cause or who had received medically prescribed treatment for more than 3 UTIs in the past 12 months, subjects suffering from immunosuppression, CNS-disorders or venereal diseases, subjects with diabetic retinopathy, nephropathy or neuropathy, subjects who currently participated in any clinical study or previously enrolled in this survey, subjects who had received treatment with an investigational product within the last 3 months and subjects who were known to be pregnant.

## Materials

The urine cultures were carried out for the purposes of this study only. Patients were required to provide a freshly voided midstream urine sample. Immediately after sampling, an Uricult dip slide was prepared and forwarded by courier to the central laboratory for microbiological testing (Laboratory for Clinical Microbiology, Central Hospital, Växjö, Sweden). No dipstick testing for leucocytes was performed in advance due to the impossibility to exclude an UTI by a negative result [17,19]. After the arrival at the central microbiological laboratory the dip slide was incubated for 18-24 hours at 35°C followed by assessing the bacterial density of the original urine sample leading to one of four possible results:

A positive result means a Gram-negative culture and ≥ 10^3^ cfu/ml, a negative result means no growth or < 10^3^ cfu/ml, mixed means the presence of more than two species, other means the growth of Gram-positive bacteria.

Significant bacteriuria was defined as ≥ 10^3^ cfu/ml as there is evidence that a significant number of women with symptoms of UTI do not have bacteriuria exceeding this value [20]. Further, this value corresponds to the lower limit of the former ECO·SENS survey [17].

Qualitative assessment was done by sub-culturing for identification and testing for antibiotic susceptibility of *E. coli* and *K. pneumonia*. The isolates were tested against the following antimicrobial agents: Mecillinam, ampicillin, amoxicillin + clavulanic acid, sulphametoxazole trimethoprim, trimethoprim/sulfamethoxazole, ciprofloxacin, nalidixic acid, nitrofurantoin, gentamicin, fosfomycin/trometamol, Cephadroxil, cefotaxime and ceftazidime. The susceptibility of *E. coli* and *K. pneumonia* is expressed by inhibition zone sizes and grouped as susceptible or resistant in accordance with the results of the disk diffusion method and the corresponding breakpoint values published by the Swedish Reference Group for Antibiotics [21]. The distribution of mecillinamin MIC-values for both *E. coli* and *K. pneumonia* isolates was determined by Etest®.

In addition to the antibiotic resistance data, demographic data (age) of the patients related were documented by the GPs. The age was stratified into two clusters: The first one with patients aged 18-50 years, the second one with patients aged 51-65 years.

### Data analysis

The prevalence of the bacteria were described first in relation to all included urine samples and then in relation to all cultures with a significant growth. Statistically significant differences in the frequency of *E.coli* in the different age subgroups were performed by using the Pearson’s Chi-Square test (alpha 5%; CI 95%). The resistance rates for *E.coli* and *K.pneumoniae* were described for each of the 14 antibiotics by conduction descriptive statistical methods.

The most frequent combinations of multi-resistances to antimicrobial agents were listed along their proportions and exact confidence intervals.

Additionally, the proportion of resistant *E. coli* isolates and exact confidence intervals were contrasted with the findings from the original ECO·SENS project. Changes between the resistance rates of the two studies were described. Moreover, these results were contrasted with the resistance data for *E. coli* in the Austrian resistance report 2008 (AURES) for the ambulatory sector to show possible differences.

### Ethical considerations

The study was approved by the ethical committee of Lower Austria (Nr.GS4-EK-4/019-2007).

## Results

### Patients characteristics

Within the study period of 71.4 weeks a total of 1,776 subjects were enrolled in the ECO·SENS II study in all five countries of which 1,697 cases could be analyzed.

**Figure 1:**
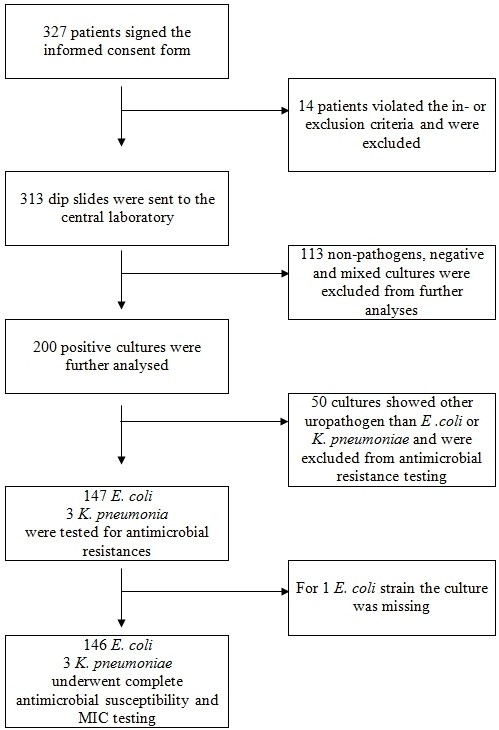
Flow chart for the recruiting, sampling and testing process for patients, urine samples and bacterial specimens.

Figure 1 shows the flow chart for the sampling and analyzing process for patients, urine samples and bacterial specimens for Austria. In Austria 23 primary care investigational centre’s recruited 327 patients of which 14 had to be refused due to the exclusion criteria. The 313 remaining patients had a mean age of 40.2 (SD 14.8) years and a mean total symptom score of 5.5 (SD 2.7). All 313 urine samples of these patients were included in the further analyses. Overall, there has been a significant bacterial growth in 64% (n = 200) of the 313 urine samples. Table 1 shows the distribution of all culture test results, first, in relation to all 313 samples and, in addition, in relation to the 200 samples with bacterial growth.

**Table 1:** Distribution of all culture test results in relation to all 313 samples and in relation to the 200 samples with bacterial growth

**Culture results**	**No. of all cultures (%)**	**No. of positive cultures (%)**
	**(n = 313)**	**(n = 200)**
*E. coli*	147 (47%)	147 (73.5%)
*K. pneumonia*	3 (1%)	3 (1.5%)
Other uropathogens*	50 (16%)	50 (25.0%)
Non-pathogens or negative or mixed^§^ cultures	113 (37%)	-

*Proteus mirabilis, Klebsiella spp., other Enterobacteriaceae, *Staphylococcus saprophyticus*, enterococci and *Pseudomonas* spp.

^§^Mixed means more than two species.

Concerning the two age cluster no statistically significant difference in the prevalence of *E. coli* in relation to all 313 samples (48.8% in age group 18-50 years vs. 44.0% in age group 51-65 years; p > 0.05) or in relation to the 200 samples with a positive culture growth (75.2% vs. 71.3%; p > 0.05) could be found.

Due to the very low number of *K. pneumonia* isolates found (n = 3) they were excluded from any further calculations.

### Resistance data

Table 2 shows the antimicrobial resistance rates of the *E. coli* isolates against 14 antibiotics. The antimicrobial resistance as described in the table was very low for mecillinam (MEC), nitrofurantoin (NIT), fosfomycin trometamol (FOS) and gentamycin (GEN), low for cefotaxime (CTX), ceftazidime (CAZ), Cephadroxil (CDR) and ciprofloxacin (CIP), higher for amoxicillin/clavulanic acid (AMC), nalidixic acid (NAL), trimethoprim/sulphamezoxazole (TSU)and trimethoprim (TRI) and highest for sulphamethoxazole (SUL) and ampicillin (AMP).

**Table 2:** **Antimicrobial resistances to 14 antimicrobial agents of the 146 *E. coli *strains**

**Antimicrobial agent**	**% (95% CI)**	(N = 146*)
Mecillinam	0.00 (0.00 - 2.49)	(n = 0)
Ampicillin	28.77 (21.58 – 36.83)	(n = 42)
Amoxicillin/clavulanic acid	8.90 (4.83 – 14.74)	(n = 13)
Trimethoprim	15.75 (10.26 - 22.69)	(n = 23)
Sulphamethoxazole	21.23 (14.91 – 28.76)	(n = 31)
Trimethoprim/Sulphamethoxazole	14.38 (9.13 – 21.14)	(n = 21)
Nalidixic acid	9.59 (5.34 – 15.57)	(n = 14)
Nitrofurantoin	0.68 (0.02 – 3.76)	(n = 1)
Ciprofloxacin	4.11 (1.52 – 8.73)	(n = 6)
Gentamicin	1.37 (0.17 – 4.86)	(n = 2)
Fosfomycin trometamol	0.68 (0.2 – 3.76)	(n = 1)
Cefadroxil	4.11 (1.52 – 8.73)	(n = 6)
Cefotaxime	2.74 (0.75 – 6.87)	(n = 4)
Ceftazidime	2.74 (0.75 – 6.87)	(n = 4)

Since there are resistances against more than one antibiotic the sum of the absolute numbers exceeds 146.

* The antimicrobial resistance result of one isolate is missing.

**Figure 2:**
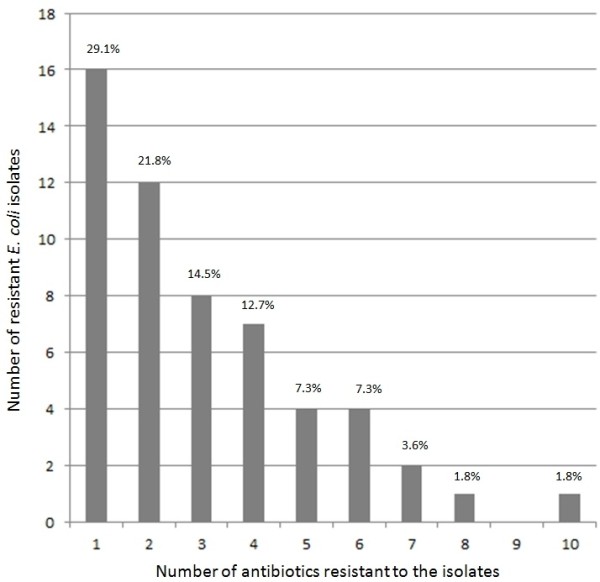
**Distribution of the 55 single and multi-resistant E. coli isolates.** The bars represent the absolute numbers of isolates in which resistance against “1” to “10” different antibiotics were observed at the same time. The percentages indicate the distribution within this sample.

In total, 91 (62.3%) of the 146 isolates were susceptible to all tested antibiotics, 16 (10.9%) isolates were resistant to one antibiotic, 12 (8.2%) were resistant to two, 8 (5.5%) isolates to three and 19 (13.1%) isolates to four and more antibiotics. Figure 2 describes the distribution of the 55 (37.7%) single and multi-resistant *E. coli* isolates. For Austria 20 different multi-resistant phenotypes were found in *E. coli*. The most common resistance types were AMP-SUL (4.11%; CI 1.52- 8.73), AMP-TRI-SUL-TSU (3.42%; CI 1.12- 7.81), TRI-SUL-TSU (3.42%; CI 1.12- 7.81), AMP-AMC (2.74%; CI 0.75- 6.87), AMP-AMC-TRI-SUL-TSU (2.05%; CI 0.43- 5.89) and AMP-TRI-SUL-TSU-NAL-CIP (1.37%; CI 0.17- 4.86). All other resistance phenotypes had an equal frequency of 0.68% (CI 0.02- 3.76).

The isolate with the highest number of resistances to different antibiotics was resistant to AMP-AMC-TRI-SUL-TSU-NAL-CIP-CDR-CTX-CAZ.

The susceptibility testing of the 146 *E. coli* isolates with mecillinam demonstrated that 50.7% had MIC values of 0.125 Mg/L and 17.8% had MIC values of 0.25 Mg/L.

Two ESBL producing isolates of *E. coli* were detected both belonging to the CTX-M-1 group. The MIC values ranged from 64 to 256 mg/L for cefotaxime and from 16 to 256 mg/L for ceftazidime. Both strains were susceptible to mecillinam with MIC values between 0.38 and 1.0 mg/l. No ESBL producing strains of *K. pneumonia* were found.

### Comparison of the results from ECO·SENS, ECO·SENS II and the results from the Austrian resistance report 2008 for *E. coli* for the ambulatory sector

Table 3 describes the differences in the resistance findings of *E. coli* against 12 antibiotics of the ECO·SENS and ECO·SENS II studies and the results from the Austrian resistance report 2008 for *E. coli* for the ambulatory sector.

**Table 3:** **Comparison of resistance rates among *E. coli *in Austria between ECO·SENS and ECO·SENS II and contrasting these data with the *E. coli *resistance data in the Austrian resistance report 2008 for the ambulatory sector**

**Antibiotic**	**Resistance rates**	**Resistance rates**	**Trend between ECO·SENS and ECO·SENS II**	***E. coli *resistance data in the Austria resistance report 2008 for the ambulatory sector, n (%)**
	**ECO·SENS**	**ECO·SENS II**		
	**(N = 126), n (%)**	**(N = 146), n (%)**		
Mecillinam	2 (1.6)	0 (0.0)	↘	4,361 (12.2)
Ampicillin	22 (17.5)	42 (28.8)	↗	8,992 (39.8)
Amoxicillin/clavulanic acid	3 (2.4)	13 (8.9)	↗	8,985 (5.8)
Trimethoprim	12 (9.5)	23 (15.8)	↗	-
Sulphamethoxazole	32 (25.4)	31 (21.3)	↘	-
Trimethoprim/Sulphamethoxazole	12 (9.5)	21 (14.4)	↗	8,992 (24.6)
Nalidixic acid	3 (2.4)	14 (9.6)	↗	-
Nitrofurantoin	1 (0.8)	1 (0.7)	↘	8,789 (2.2)
Ciprofloxacin	0 (0.0)	6 (4.1)	↗	8,992 (15.7)
Gentamicin	1 (0.8)	2 (1.4)	↗	8,990 (4.7)
Fosfomycin trometamol	0 (0)	1 (0.7)	↗	5,489 (1.5)
Cefadroxil	1 (0.8)	6 (4.1)		9,088 (8.5)
Cefotaxime	Not tested	4 (2.7)		-
Ceftazidime	Not tested	4 (2.7)		-

An increase in microbial resistance against ampicillin, amoxicillin/clavulanic acid, nalidixic acid and ciprofloxacin could be observed between the study period of the ECO·SENS study in 1999/2000 and the ECO·SENS II study in 2007/2008.

By contrasting the results of the ECO·SENS II study with the results from the Austrian resistance report 2008 for the ambulatory sector, the resistance results in the Austrian resistance report 2008 were much higher. Ampicillin (39.8%), trimethoprim/sulphamethoxazole (24.6%) and ciprofloxacin (15.7%) were the AB with the highest resistance rates for *E. coli* but also mecillinan (12.2%), Cephadroxil (8.5%) and nitrofurantoin (2.2%) showed much higher resistance rates compared to the ECO·SENS II study.

## Discussion

The resistance data for *E. coli* in uncomplicated UTIs in 18-65 year old women gained by this study are the most recent data for this disease in Austria at this moment [12]. Moreover, Austria participated in both surveys in the ECO·SENS 1999/2000 as well as in the ECO·SENS II 2007/2008. For this reason changes in the antibiotic resistances could be described between these two periods and interpreted with caution. The chance to conduct such a comparison was due to the number given by the centre’s (19/23), the number of patients recruited (298/327), the mean age (41.0/40.2) and the symptom score (5.4/5.5) were similar and the inclusion/exclusion criteria as well as the study procedures, the laboratory and the susceptibility testing according to the Swedish Reference Group for Antibiotics and its Subcommittee on Methodology (SRGA) [21] for the two studies were identical. Nevertheless, it has to be considered that this comparison is between two cross-sectional studies and, therefore, it has limitations which were discussed below.

The study showed that 47% of women complaining of symptoms of an uncomplicated UTI had significant bacteriuria caused by *E. coli* which is 73.4% of all grown bacteria; a percentage very similar to the results found in the ECO·SENS study nine years before and the ARESC study from 2006 [16,17].

Concerning the resistance patterns of *E. coli* the study showed that mean resistance rates of ampicillin (28.8%), sulphametoxazole (14.4%), trimethoprim (15.8%) and trimethoprim/sulphametoxazole (14.4%) remained high or even increased (ampicillin) between the years 1999/2000 and 2007/2008. Lower mean resistance levels were found for nalidixic acid (9.6%), amoxicillin/clavulanic acid (8.9%), ciprofloxacin (4.1%) and Cephadroxil (4.1%), with a change (except Cephadroxil) in resistance rates between the two ECO·SENS studies. Resistance rates of mecillinam (0%), nitrofurantoin (0.7%), fosfomycin (0.7%), gentamycin (1.4%), cefotaxime (2.7%) and ceftazidime (2.7%) remained very low. Based on the literature we could confirm the persisting very low resistance rate for mecillinam [8].

Fortunately, the resistance of *E. coli* to nitrofurantoin and fosfomycin were both less than 1% since these two antibiotics are recommended in the S3-guideline for the treatment of uncomplicated UTI as first-line drugs [22] but how is the prescribing behavior of GPs and what do local guidelines recommend? For instance the Austrian ABSGROUP recommend pivmecillinam, amoxicillin/clavulanic acid and sultamicillin as first-line drugs [23]. For amoxillin/clavulanic acid the resistance rate in the ECO·SENSE II study for Austria was 8.9% which is not yet alarming but a negative trend. Therefore, our study can promote further surveillance of antibiotic resistance, especially, in the primary health care sector not only to check the currentness of guidelines but also the make the GPs think about their own prescribing behavior, not only in Austria.

Only 2 (1.4%) ESBL producing isolates of *E. coli* and no ESBL producing strain of *K. pneumonia* were found which is comparable to a study from France with low level of ESBL (1.2%) in uncomplicated UTIs in outpatients [24].

The quite high and compared to the results of the ECO·SENS study increased resistance rates of amoxicillin/clavulanic acid, ciprofloxacin and nalidixic acid should be respected when choosing an appropriate antibiotic for uncomplicated UTIs. The use of ampicillin, sulphamethoxazole, trimethoprim and trimethoprim/sulphametoxazole in uncomplicated UTIs in women should be questioned as suggested by the even higher resistance results of the ARESC-study (48.3% for ampicillin and 29.0% for trimethoprim/sulphametoxazole) [16].

The increase in fluorochinolon resistance is not yet alarming but deserves further careful observation taking into consideration the increasing use of this antibiotic in uncomplicated UTIs [25,26].

The comparison of the resistance rates of this study with the corresponding rates of the Austrian Resistance Report 2008 (AURES) shows much higher resistance rates for aminopenicillin, fluorochinolon and trimethoprim/sulphametoxazole as well as a higher rates to mecillinam in the AURES 2008 [15]. The reason for the higher resistance rates described in the AURES 2008 may result from the different data collection methodology. In the AURES the resistance data for all *E. coli* specimens sent to one of the reference laboratories are described. In a routine primary health care setting, samples from the primary care sector are sent predominantly to a laboratory if a complicated UTI occurred. Therefore, these data do not fit as reference data for local or national guidelines. However, in a lot of countries data collected like these are the only available which should be questioned. To obtain comprehensive resistance data for uncomplicated UTIs that have to find their way into the guidelines, clearly structured studies have to be performed on a regular base.

The missing resistance testing of uropathogens like other *Enterobacteriaceae* and *Staphylococcus saprophytic us* as well as the low numbers of *K. pneumonia* isolates and the exclusion of mixed cultures can be considered as limitation of this study. Further limitations were the patients and GPs inclusion process which was consecutive and voluntary. It could be speculated that the GPs that were already sensible concerning the topic antibiotic resistance and prudent antibiotic prescription participated in this study only. Moreover, the comparison of the results of two cross-sectional studies has to be questioned due to the lack of knowledge concerning further demographic data of the participating patients and the non-exact matching of the participating GP offices.

## Conclusions

The resistance data found are an important contribution to the therapeutic safety and quality in General Practice in Austria.

Since there is a growing concern about an increase in antibiotic resistance also in the ambulatory sector due to frequent and sometimes uncritical prescriptions of antibiotics the results of this study could help to estimate the scope of the problem by increasing the knowledge about antibiotic resistance pattern before a possible therapy is prescribed. The knowledge of changes in antibiotic resistance over time is the next essential step for choosing the appropriate antibiotic [27]. The findings of this study should result in a regular surveillance system for bacteria and resistances emerging in the ambulatory sector designed after the model of the EARS-Net (formerly EARSS, European Antimicrobial Resistance Surveillance System) [28] for women and for men. It is not enough to sample and test all specimens coming to a laboratory from the ambulatory sector by chance to obtain comprehensive resistance data for the primary health care sector for special diseases. Structured studies have to be performed on a regular base. In addition to a surveillance system, more UTI preventive interventions should be promoted [29].

## Competing interests

The first, fourth and fifth author participated in this study as GPs.

The authors declare that they have no competing interests.

## Authors’ contributions

GK was the national coordinator of this multicentre study. He prepared and drafted the initial manuscript. GW reviewed the literature and participated in drafting the reference. He revised the manuscript critically with focus on methods. Furthermore, he drafted the abstract together with KH. SZ performed the statistical analyses and critically revised the interpretation of data. WF participated in drafting the initial manuscript and gave input concerning the relevance of the results for primary care. WS participated in revising the first draft of the manuscript and by searching for relevant literature. KH reviewed the literature and critically revised the manuscript and references. Furthermore, she drafted the abstract together with GW. All authors read and approved the final manuscript.

## Pre-publication history

The pre-publication history for this paper can be accessed here:

http://www.biomedcentral.com/1471-2334/12/222/prepub
